# Subjects with carotid webs demonstrate pro-thrombotic hemodynamics compared to subjects with carotid atherosclerosis

**DOI:** 10.1038/s41598-024-60666-7

**Published:** 2024-05-02

**Authors:** Retta El Sayed, Carissa J. Lucas, Hannah L. Cebull, Fadi B. Nahab, Diogo C. Haussen, Jason W. Allen, John N. Oshinski

**Affiliations:** 1 Department of Biomedical Engineering, Georgia Institute of Technology, Emory University, 1364 Clifton Rd, Atlanta, GA 30322 USA; 2 Department of Radiology and Imaging Sciences, Emory University, Atlanta, GA USA; 3 Department of Neurology, Emory University, Atlanta, GA USA; 4 Department of Radiology and Imaging Sciences, Indiana University, Indianapolis, IN USA

**Keywords:** Carotid artery disease, Biomedical engineering, Stroke, Risk factors

## Abstract

Carotid artery webs (CaW) are non-atherosclerotic projections into the vascular lumen and have been linked to up to one-third of cryptogenic strokes in younger patients. Determining how CaW affects local hemodynamics is essential for understanding clot formation and stroke risk. Computational fluid dynamics simulations were used to investigate patient-specific hemodynamics in carotid artery bifurcations with CaW, bifurcations with atherosclerotic lesions having a similar degree of lumen narrowing, and with healthy carotid bifurcations. Simulations were conducted using segmented computed tomography angiography geometries with inlet boundary conditions extracted from 2D phase contrast MRI scans. The study included carotid bifurcations with CaW (n = 13), mild atherosclerosis (n = 7), and healthy bifurcation geometries (n = 6). Hemodynamic parameters associated with vascular dysfunction and clot formation, including shear rate, oscillatory shear index (OSI), low velocity, and flow stasis were calculated and compared between the subject groups. Patients with CaW had significantly larger regions containing low shear rate, high OSI, low velocity, and flow stasis in comparison to subjects with mild atherosclerosis or normal bifurcations. These abnormal hemodynamic metrics in patients with CaW are associated with clot formation and vascular dysfunction and suggest that hemodynamic assessment may be a tool to assess stroke risk in these patients.

## Introduction

Carotid artery webs (CaW) are shelf-like projections into the internal carotid artery representing a form of fibromuscular dysplasia that cause focal non-significant luminal narrowing^1–4^. CaW may account for up to one-third of cryptogenic strokes in younger patients between the ages 30–48 without vascular risk factors^2–5^. CaW has also been associated with recurrent ischemic stroke, but unlike carotid atherosclerosis, CaW is not characterized by inflammation, or plaque deposition but instead is composed of intimal hyperplasia^6,7^. The etiology of CaW remains elusive and is not fully understood. Additionally, the treatment of patients with CaW varies among medical centers and may include endarterectomy, carotid artery stenting, or medical management with anticoagulation, single or dual antiplatelet therapy^2,8^. A recent study suggested that medical treatment alone is often ineffective^9^.

Computed tomography angiography (CTA)^1,2,8,10^ and digital subtraction angiography (DSA)^11,12^ have been the primary modalities for clinical diagnosis of CaW, although other modalities have been investigated^5,13^. Studies suggest that CaW induces changes in the local hemodynamic parameters, causing significant disruptions in blood flow patterns^11,14–16^. The unique shape of CaW leads to flow disturbances such as flow separation, recirculation, and stagnation, which ultimately lead to the formation of thrombus. Understanding the quantitative hemodynamic alterations caused by CaW appears to be crucial for assessing the risk of thrombus formation and subsequent stroke^11,14,16,17^.

Traditionally, blood clot formation is attributed to factors in Virchow’s Triad, encompassing vessel wall damage, blood stasis, or the presence of hypercoagulability^18^. In individuals with CaW, the extent of luminal narrowing, the angle of the web, the vascular flow rate, and other geometric and hemodynamic factors may determine the level of flow disturbance and the presence of flow stasis downstream of the CaW^14,15,19,20^. Because of the significant variation in geometries among subjects, the prediction of thrombus-prone regions based on hemodynamics is multi-factorial and needs investigation on a patient-specific basis.

The goal of this study is to understand the effect of CaW geometry on local hemodynamic disturbance and its relationship to complex blood flow responsible for thrombus formation. Hemodynamics in the carotid artery has been studied previously using 4D flow MRI^16,21–23^, 2D phase contrast MRI^24,25^, computational fluid dynamics (CFD)^26,27^, and other modalities^28–30^. However, only limited studies have investigated hemodynamic disturbances related to CaW^11,15,16,31^. The current study evaluated oscillatory shear index (OSI) as a hemodynamic factor related to vessel wall vascular dysfunction/damage; and assessed shear rate and flow stasis as hemodynamics parameters related to clot formation^32,33^. Shear rate is considered an important hemodynamic parameter in clot formation, and low shear values have been linked to activation of the coagulation cascade^33–35^. Stasis is one of Virchow’s triad factors for clot formation making it an important parameter to study. OSI is a vessel wall index of the temporal variation in arterial wall shear stress (WSS) due to pulsatile flow within a cardiac cycle^36^. Low shear rate, high OSI, larger low velocity regions, and longer periods of stasis are typically considered markers of flow disturbances related to thrombus formation^37^.

We hypothesized that CaWs will be associated with complex flow regions represented by low shear rate and high OSI, as well the presence of large low velocity regions for extended periods during the cardiac cycle linked to stasis. We anticipate that regions of complex flow will be larger in CaW subjects compared to subjects with mild atherosclerotic lesions or normal subjects.

## Material and methods

### Patient population and image acquisition

This was an IRB-approved study by Emory University IRB Office (IRB number: IRB00091421) and written informed consent was obtained from all participants. All methods were performed in accordance with the relevant guidelines and regulations and the Health Insurance Portability and Accountability Act (HIPPA) guidelines were followed during the study. The study population included three groups of carotid bifurcations. Group 1 was a set of carotid bifurcations segmented from patients with CaW (number of bifurcations = 13, Sex: 3 male and 7 female, age: 51.2 ± 10.2 years). All subjects in group 1 had a history of stroke or transient ischemic attack (TIA). Subjects in group 1 had mild luminal narrowing (30 ± 11%) caused by the web, based on the ECST criteria^38^. Group 2 was a set of carotid bifurcations segmented from subjects with mild atherosclerosis (number of bifurcations = 7, Sex: 2 male and 5 female, age: 70 ± 8.3 years). Subjects in group 2 had a luminal narrowing of 42 ± 17%^38^. Group 3 was a set of normal bifurcations. Six of the CaW subjects had a unilateral web, and the side *without the web* was used as normal geometries (number of bifurcations = 6, Sex: 2 Male and 4 female, age: 54 ± 9.8). Table 1 lists participant characteristics. All subjects underwent a CTA with a spatial resolution of 0.49 × 0.49 × 0.62 mm^3^. Patient blood pressure and heart rate were measured during a clinical visit. All subjects were also imaged on a 3T MRI system (MAGNETOM Prismafit, Siemens Medical Solutions). Two-dimensional, ECG-gated, cine phase contrast (2D PCMR) images were acquired 10 mm below the carotid artery bifurcation (1 × 1 × 5 mm^3^, VENC = 80 cm/s, TR = 43.6, TE = 7). The 2D PCMR images were analyzed using the freely available software Segment (Version: 4.0 R12067; Medviso, segment.heiberg.se)^39^. The flow rate was extracted from the 2D PCMR data at the plane at the common carotid artery (CCA) and was used as an inlet boundary condition for the CFD simulation. The inlet velocity profile in the CCA 2D PCMR data was nearly parabolic, therefore a parabolic velocity profile was used in the CFD simulations as the inlet velocity profile.

**Table 1:** Summarizes subject parameters including sex, age, blood pressure, heart rate, and cardiac period (mean values and the standard deviations across subject groups are reported and (*) shows measurements acquired during a clinical visit).

	CaW (*p* value vs. atherosclerosis)	Atherosclerosis (*p* value vs. normal)	Normal (*p* value vs. CaW)
Number of carotid geometries	n = 13	n = 7	n = 6
Sex	3 Male|10 Female	2 Male|5 Female	2 Male|4 Female
Age (years)	51.2 ± 10.2 (0.002)	70 ± 8.3 (0.03)	54.0 ± 9.8 (0.66)
Systolic pressure (mm Hg) *	126.9 ± 11.8 (0.32)	135.4 ± 13.2 (0.13)	123.5 ± 14.9 (0.46)
Diastolic pressure (mm Hg)*	73.6 ± 10 (0.86)	72.3 ± 5.8 (0.13)	81.3 ± 7.8 (0.13)
MAP (mm Hg)	91.4 ± 8.5 (0.72)	93.3 ± 5.9 (0.66)	95.2 ± 9.9 (0.40)
CCA Inlet area (cm)	0.3 ± 0.1 (0.65)	0.3 ± 0.1 (0.72)	0.3 ± 0.1 (0.99)
Cardiac period (s)	0.9 ± 0.1 (0.68)	0.8 ± 0.1 (0.68)	0.9 ± 0.1 (0.95)
Heart rate*	67.2 ± 10.5 (0.69)	70.1 ± 8.4 (0.71)	65.8 ± 8 (0.97)

*p* value of each parameter was listed below the measurement in round parentheses.

### Computational fluid dynamic simulation

**Figure 1:**
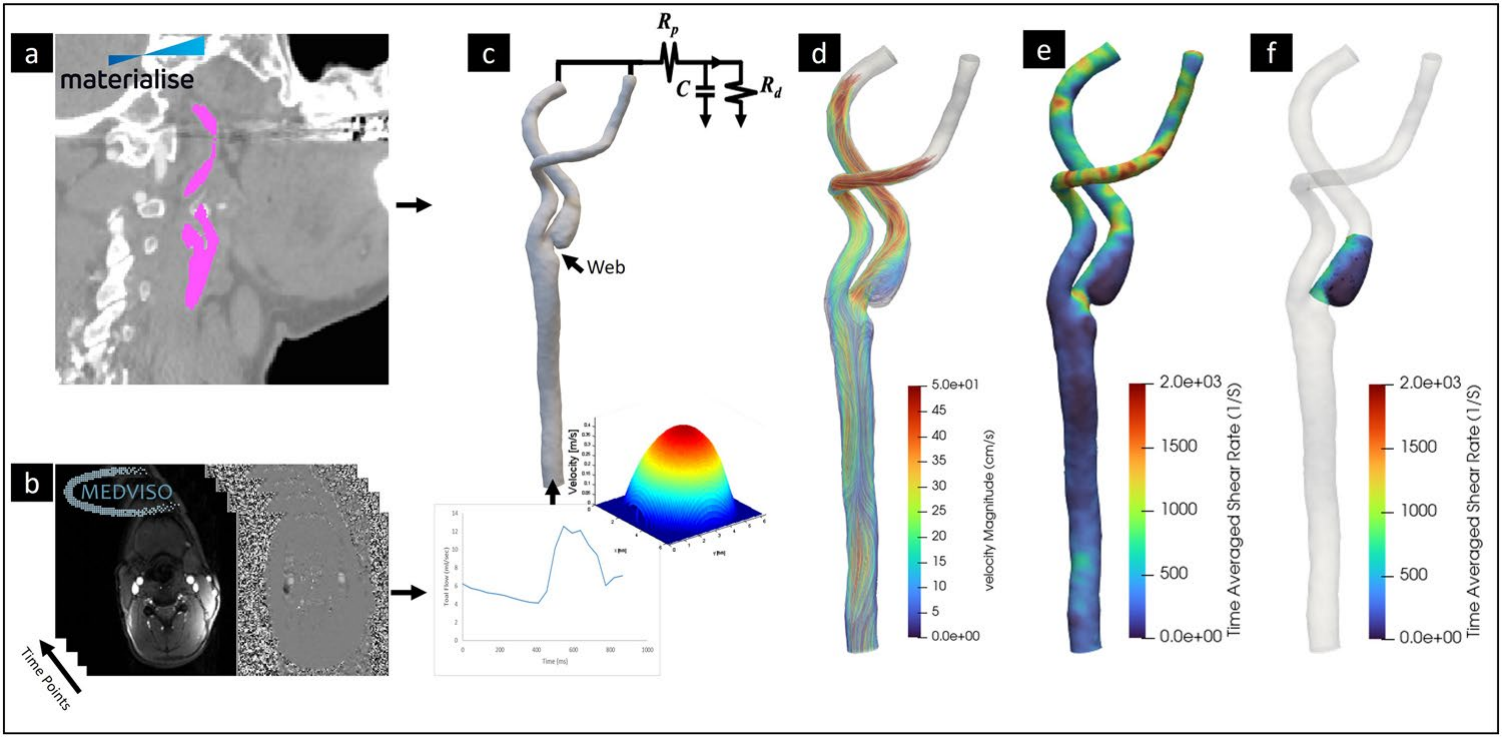
Geometry preparation and the CFD pipeline. (**a**) CTA scan loaded into Mimics Materialise and the resulting segmentation for the carotid artery in that slice. (**b**) 2D PC-MR scan loaded into Segment Medviso Software and the resulting flow waveform. (**c**) The segmented and the meshed carotid artery with CaW and the inlet/outlet boundary conditions. (**d**) Color-coded velocity streamlines and (**e**) time-averaged shear rate at the wall. (**f**) The bulbar segment is selected where the complex flow is expected.

Mimics Materialise NV (version: 22.0.0.524) was used to segment the carotid artery from the cross-sectional CTA images of all subjects (Fig. 1). The 3D geometry was then imported in 3-Matics Materialise NV (version: 22.0.0.524) for removing small branches, artifacts, minor surface smoothing and initial meshing. The STL file exported from 3-Matics was imported into SimVascular (Version: 2022.07.20) for geometry meshing and running a transient flow simulation^40,41^. The geometry meshing was done as a tetrahedral finite element adaptive mesh (TetGen open-source mesh generator). To ensure that our hemodynamics were not affected by the mesh resolution, a mesh-independence study was conducted. The mesh sizes of 0.04, 0.045, 0.05, 0.06, and 0.07 mm were tested; the velocity and pressure of a probe located in the common carotid were compared. We determined that 0.05 mm was the largest element size that did not decrease accuracy and minimized computational costs^41^. Increasing mesh density near the vessel wall is helpful when large velocity and pressure gradients are expected, therefore boundary layers were added^40^. To determine the optimal number of boundary layers, the number of layers varied in a similar fashion (0, 1, 3, 5, and 10)^40^. Three boundary layers with mesh size decrease ratio of 0.8 provided an optimal setting for a higher velocity gradient near the wall^40,41^. The number of nodes, elements, edges, and faces averaged across each subject group is shown in Supplementary Table S1. Subject-specific mean arterial pressure (MAP) was computed using patient-specific systolic and diastolic pressure (Table 1). MAP was applied as initial pressure, and five cardiac cycles were run to ensure stable pressure. The 5th cycle was chosen to represent the solution. The fluid was modeled as a Newtonian fluid with a density of 1.06 g/cm^3^ and a viscosity of 0.04 Poise. Flow waveforms exported from 2D PCMR were used as inlet boundary conditions, assuming a parabolic velocity profile. The vessel walls were assumed to be rigid with no-slip conditions. Subject-specific resistance and capacitance values were estimated and applied to the outlets using the RCR Windkessel Model Supplementary Table S1. Capacitance was computed as the stroke volume divided by the pulse pressure and resistance was computed by the ratio of MAP to the mean flow with Murray’s Law Coefficient assumed to be 2.1 (Fig. 1)^40^. Averaged values between subjects of the total resistance and capacitance are seen in Supplementary Table S1. Supplementary Table S2 in the supplemental material has a complete list of the parameters used for the CFD simulations. The results were visualized and quantified in ParaView (Version 5.11.1)^42^. Figure 1d,e shows a typical result and representation of the solution including velocity streamlines and shear rate results.

### Region of interest (ROI)—carotid artery bulbar segment

Complex flow patterns will be present downstream of the web^30,43^. Therefore an volumetric ROI was defined in the ICA bulb and specified to be the region extended longitudinally from the narrowest point of the lumen in the web or atherosclerotic lesions (or the bifurcation in the normal geometry) to 1.5 CCA diameters downstream, where complex flow is expected to dissipate Fig. 1f^43^. To segment the ROI region, the Vascular Modeling Toolkit (VMTK) was used in Python (version 3.9.16)^44^. The centerline of the vessel, its branches, and the bifurcation point were extracted using VMTK functions^44^. Additionally, the centerline was precisely clipped at the designated ROI, ensuring consistency and eliminating the need for manual segmentation of the ROI.

### Computation of hemodynamics parameters

SimVascular was used to post-process the simulation output and extract hemodynamic parameters including localized time-dependent velocity, pressure, wall shear stress (WSS), as well as time-averaged WSS (TAWSS) and oscillatory shear index (OSI)^40^. Low shear and flow stasis, markers of prothrombotic regions, were calculated from the SimVascular output as described below.

#### Low shear regions

To determine the shear rate at the vessel wall, the WSS was divided by the blood viscosity used in the simulation (0.04 Poise). The threshold for defining ‘low shear’ in the bulb ROI regions was determined based on a literature review and a threshold sensitivity analysis. Based on a literature review, a shear rate below a value of 10 s^−1^ has been linked to coagulation and clot formation^34,35,45^. This threshold was also close to the lowest 1% of all subjects’ time-averaged shear rate data combined (8.11 s^−1^). The percentage area of the low shear rate within the bulb ROI was quantified using the surface area of the ROI that was below the low shear threshold of 10 s^−1^ divided by the surface area of the entire ROI. The low shear area was quantified across all cardiac timeframes, in addition to quantifying the time-averaged values.

#### High OSI regions

OSI was quantified automatically in the SimVascular output^40^. There is no consensus of a pathologic cut-off for high OSI in the literature, so we adopted a 1% threshold by combining all subject data and taking the top 1% of all subjects’ OSI data.

#### Low velocity-regions

The velocity output from SimVascular was imported to ParaView (Version 5.11.1)^42^, and only voxels under a specific threshold would be included as regions of low velocity regions. Since there is no consensus on a pathological threshold consistent with thrombus formation, multiple thresholds and a sensitivity analysis were conducted to determine a threshold for low velocity regions. Thresholds of 0.5, 1, 2, 3, 5, 7.5, and 10 cm/s were evaluated, and the volume of the low velocity regions was compared to determine which threshold best separated CaW bifurcations from atherosclerotic and healthy bifurcations^46^.

#### Blood stasis

To determine how long the low velocity regions are present during the cardiac cycle, “flow stasis” was quantified. Stasis in the CFD literature has been quantified using many methods such as the injection of dye and calculating the washout time^47^, calculating relative residence time (RRT) as the inverse function of TAWSS and OSI^37^, or by computing a passive scalar representing the blood residence time^48^. In this study, stasis was quantified as the percentage of time in the cardiac cycle exposed to low velocity regions under the determined threshold (number of frames with low velocity/number of total frames*100)^46,49^.

#### Statistical analysis

All quantities are represented as a mean averaged in each subject population with standard deviation. All statistical analyses were performed using IBM SPSS Statistics software (v.29 Chicago, SPSS Inc). Since the number of patient-specific geometries with atherosclerosis and normal geometries are each less than n = 10, the independent-samples Kruskal–Wallis test was used as a non-parametric (non-Gaussian distribution) analysis to determine statistical significance by pairwise comparisons of group type and *p* value < 0.05 was used as a significance level. Furthermore, ROC classifier analysis was conducted for threshold sensitivity statistical analysis. ROC curves, the area under the ROC curve, and the Gini index of the classifier metrics with the model quality measurement were quantified.

## Results

**Figure 2:**
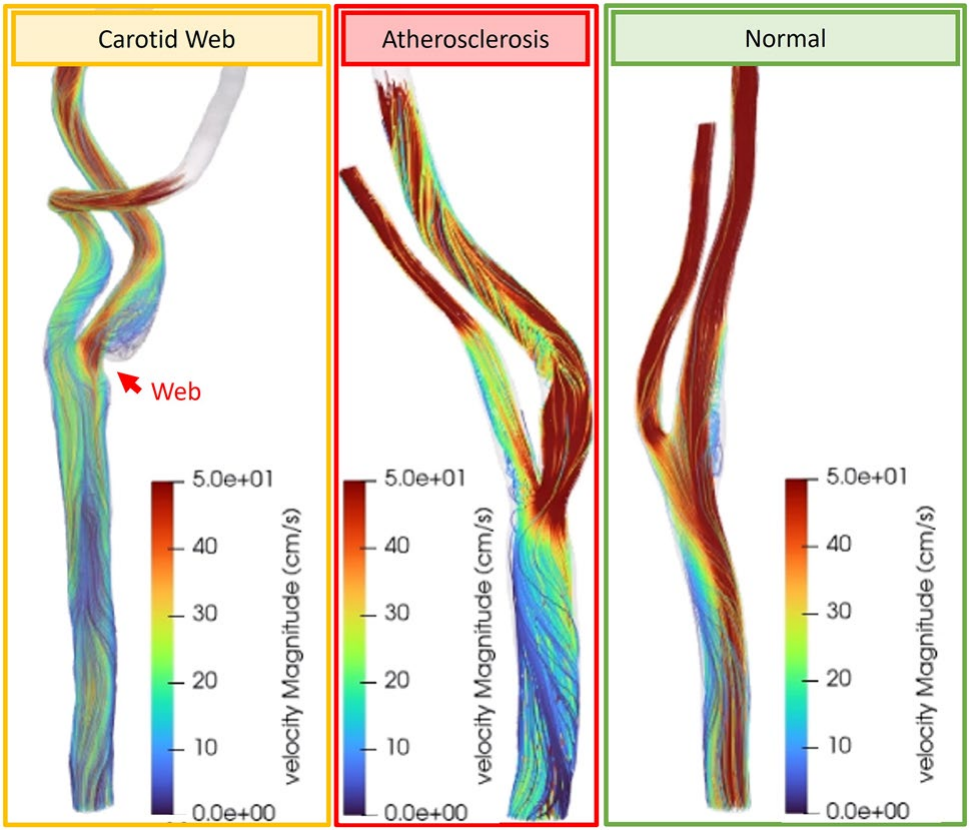
Shows an example geometry of (**a**) a carotid web, (**b**) a carotid bifurcation with mild atherosclerosis, and (**c**) a normal carotid bifurcation.

**Figure 3:**
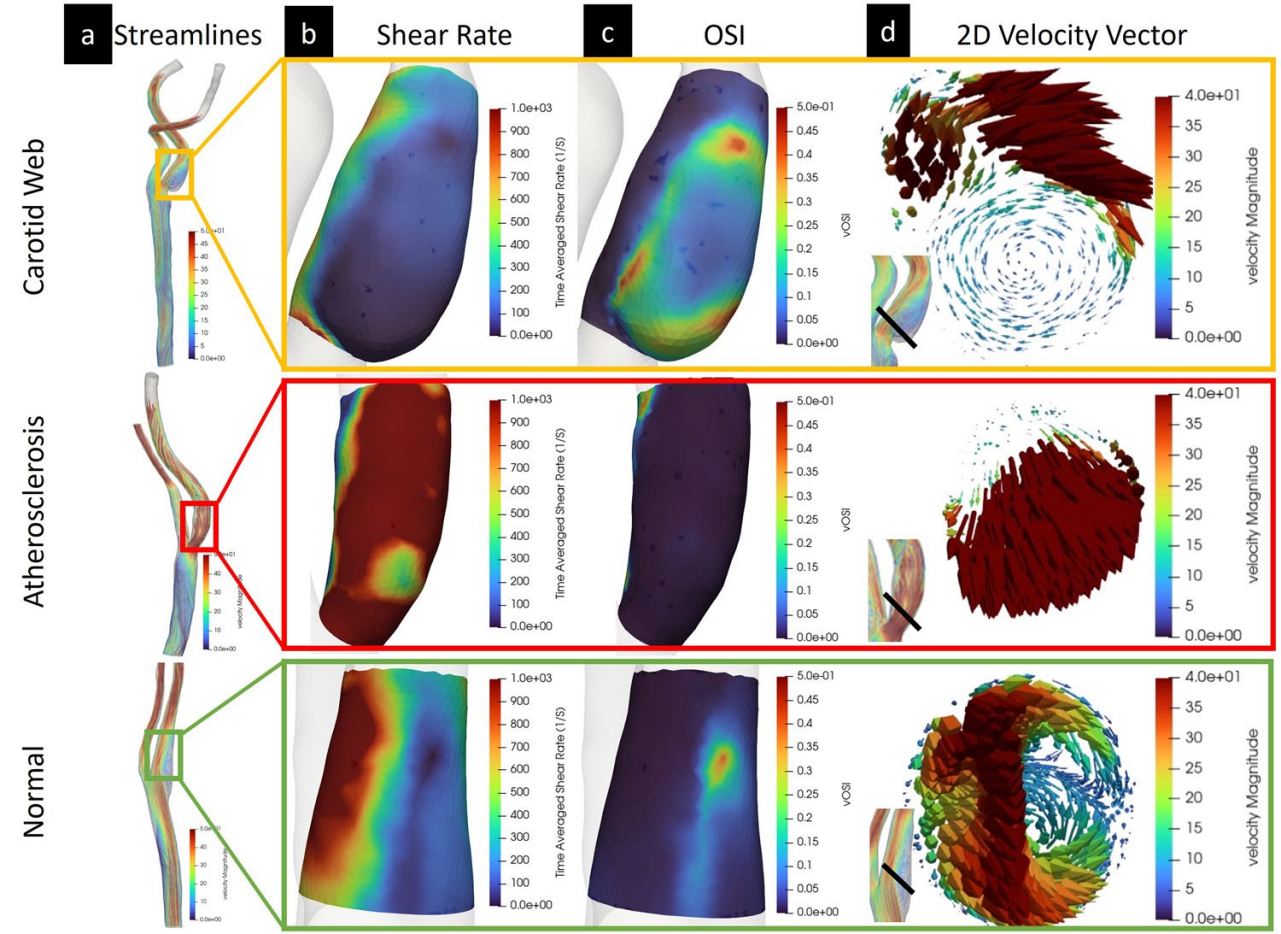
Qualitative analysis of selected carotid web, mild atherosclerosis, and normal subject-specific geometry CFD solution. (**a**) Color-coded velocity streamlines over the entire computational domain, (**b**) time-average wall shear rate in the bulb ROI segment, (**c**) OSI in the bulb ROI segment, and (**d**) 2D cross-section of a velocity vector at peak systole in the carotid artery bulb ROI segment at the plane shown.

Selected cases of velocity streamlines in a bifurcation with CaW, a bifurcation with mild atherosclerosis, and a normal bifurcation are shown in Fig. 2. Visually, subject-specific geometries with CaW show large regions exposed to complex flow patterns represented by larger areas with low shear rates and high OSI (Fig. 3b,c). The velocity vector view of a 2D cross-sectional plane at peak systole in the bulbar segment shows a flow jet on the interior wall of the bifurcation, and a region of flow separation/recirculation in the subject with CaW (Fig. 3d).

### Low shear regions

**Figure 4:**
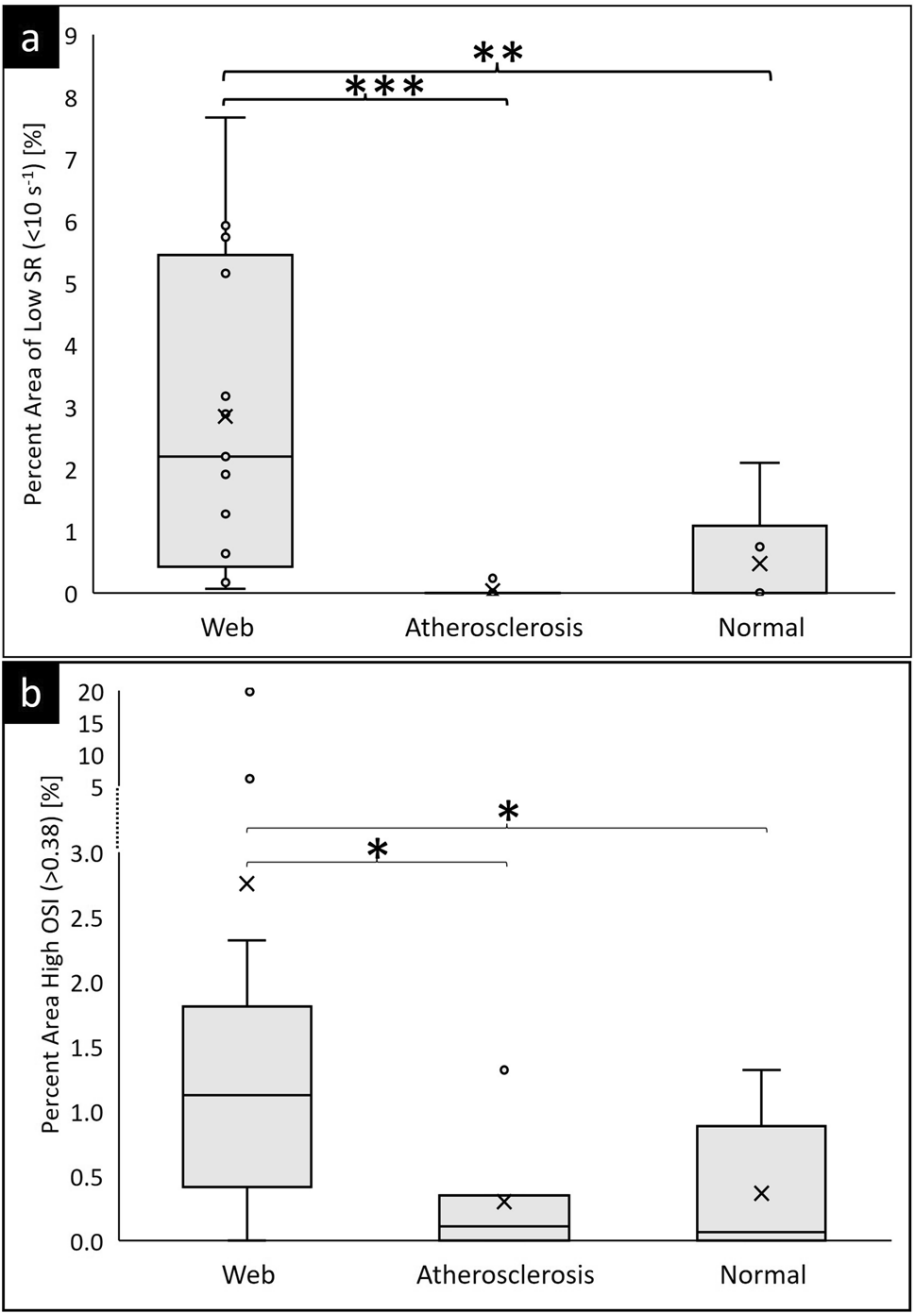
Box and whisker plots show the quantitative results for hemodynamic parameters in the carotid bulb ROI. (**a**) Percent of carotid bulb ROI surface area exposed to low shear rate and (**b**) high OSI in subjects with CaW (n = 13), subjects with atherosclerosis (n = 7), and normal subjects (n = 6). (*), (**), and (***) indicates statistically significant results of *p* value ≤ 0.05, ≤ 0.01, and ≤ 0.001, respectively.

Based on a threshold of 10 s^−1^, the low shear rate regions were quantified in the ROI. Figure 4a shows the quantitative results of a low shear rate in the carotid bulbar segment ROI across the different groups. Patients with CaW have a significantly larger region of low shear rate area (2.84 ± 2.43%) compared to subjects with mild atherosclerosis (0.03 ± 0.03%; *p* value compared to CaW < 0.001) or normal subjects (0.47 ± 0.77%; *p* value compared to CaW 0.01).

### High OSI regions

The top 1% of OSI values across all subjects was 0.38 (a scale of 0–0.5). Figure 4b illustrates that CaW subjects have a larger surface area where high OSI is present compared to mild atherosclerosis and normal subjects. Patients with CaW have a significantly larger region of high OSI (2.76 ± 5.16%) compared to subjects with mild atherosclerosis (0.30 ± 0.43%; *p* value compared to CaW 0.05) or normal subjects (0.36 ± 0.50%; *p* value compared to CaW 0.04).

### Low velocity regions

**Figure 5:**
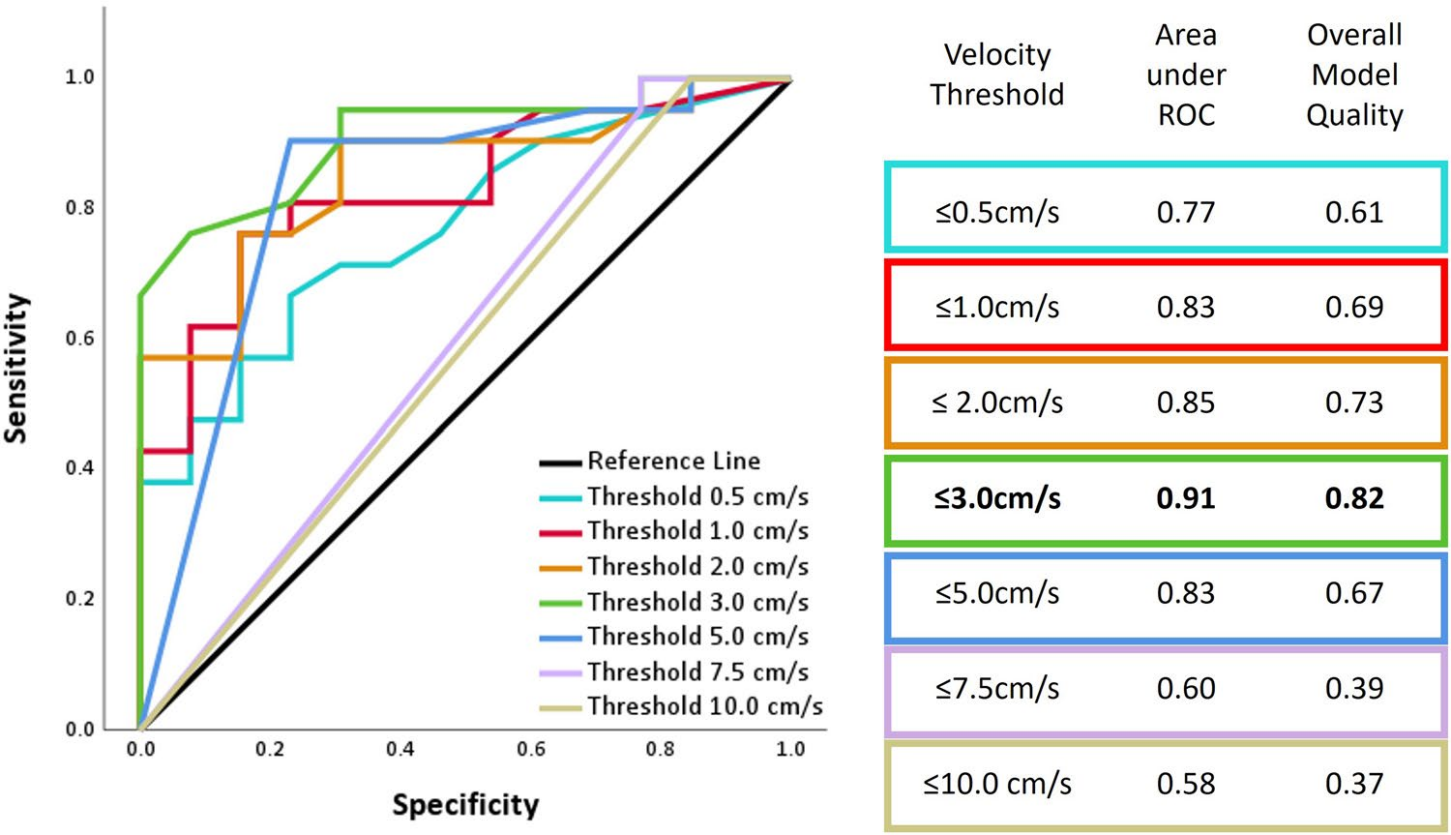
Sensitivity study analysis in subjects with a TIA or stroke event (subjects with CaW n = 13) and subjects without an event (subjects with atherosclerosis and normal n = 13). ROC curve as a classifying analysis to determine the optimal low velocity threshold with a table showing the area under the ROC curve and the overall model quality.

Since there is no consensus value for low velocity required for thrombus, a threshold sensitivity study was conducted to determine the optimal threshold for low velocity regions that best separated the CaW subjects from atherosclerotic and normal subjects. The threshold sensitivity study was conducted using ROC classifier model showing the performance at different thresholds (0.5–10 cm/s) (Fig. 5). Since all the CaW subjects had a TIA or stroke event, they were classified as positive for an event, while subjects with mild atherosclerosis and normal carotid bifurcations were considered as negative for an event. In Fig. 5, a threshold of 3cm/s showed the largest area under the ROC curve and the best overall model quality. Therefore, this value was then used to quantify the size of the low velocity regions.

**Figure 6:**
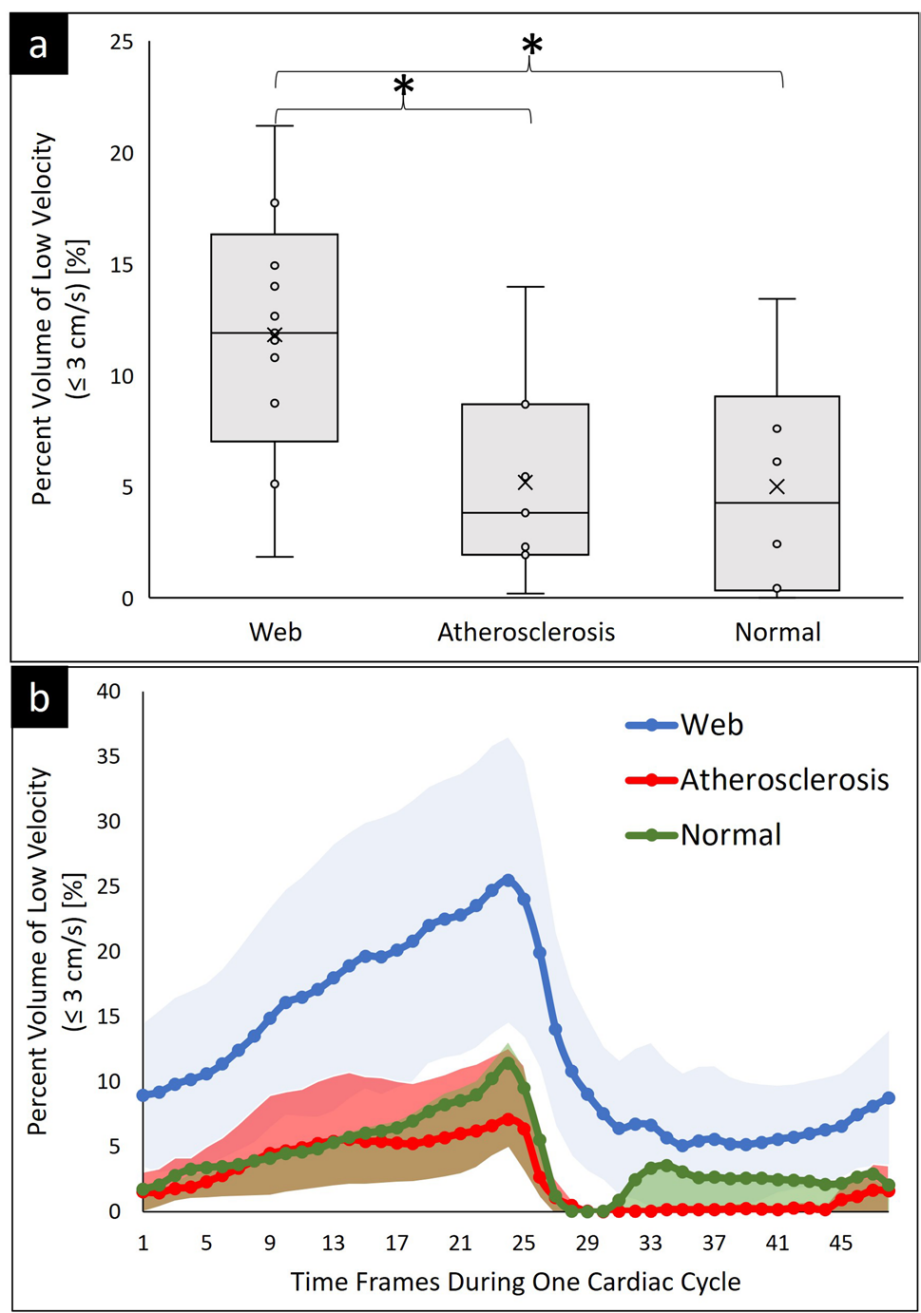
Volume of low velocity regions in CaW (n = 13), atherosclerosis (n = 7), and normal carotid bifurcation (n = 6). (**a**) Box and whisker plot shows the percent volume of low velocity (≤ 3cm/s) regions averaged across the different subject populations. (**b**) Percent volume of low velocity regions as a function of time over the cardiac cycle in each subject group. The light shading around the waveform is the standard deviation around the subjects. (*) shows statistically significant results of *p* value ≤ 0.05.

Figure 6a shows the low velocity regions as percent volume with a threshold of 3cm/s in the bulb ROI segment of the carotid bifurcation. Patients with CaW have larger regions of low velocity (11.8 ± 5.4%) compared to subjects with atherosclerosis (5.2 ± 4.4%; *p* value compared to CaW 0.03) and normal subjects (5.0 ± 4.7; *p* value compared to CaW 0.03) (Table 2).

**Table 2:** Shows a summary result of the stasis and low velocity regions quantified in the carotid artery bulbar segment; the *p* value of each parameter was listed below each parameter measured in round parentheses:

	CaW (*p* value vs. atherosclerosis)	Atherosclerosis (*p* value vs. normal)	Normal (*p* value vs. CaW)
Volume of low velocity Region [%] Threshold: 3 cm/s	11.8 ± 5.4 (0.03)	5.2 ± 4.4 (0.96)	5.0 ± 4.7 (0.03)
Stasis [% of cardiac cycle] Threshold: 3 cm/s	95 ± 12 (< 0.001)	58 ± 10 (0.38)	84 ± 10 (0.02)
Number of subjects exposed to low velocity across 100% of the cardiac cycle	9/13	0/7	0/6

### Blood stasis

To examine the time-dependent behavior of hemodynamics, we analyzed low velocity regions as a function of the percent of the cardiac cycle and refer to this as stasis. Figure 6b illustrates the volume of the low-velocity region computed for each cardiac time frame and subsequently averaged within each subject group (CaW, atherosclerosis, and normal). The standard deviation between subjects is highlighted on the figure to provide insights into subject variability. Grouping all subjects with CaW (Fig. 6b), the *average* velocity within regions in the carotid bulb segment remain consistently below the 3 cm/s threshold throughout the entire cardiac cycle. This is in contrast to subjects with atherosclerosis or normal subjects where the velocity rises above this threshold value at some point in the cardiac cycle to clear and flush out the bulb in all subjects.

Table 2 contains quantitative results of the percent volume of the low velocity (threshold < 3cm/s), and the corresponding stasis as a percentage of time in the cardiac cycle averaged across each subject group. Subjects with CaW contain significantly higher stasis (95 ± 12%) compared to atherosclerosis (58 ± 10; *p* value compared to CaW < 0.001) and normal subjects (84 ± 10; *p* value compared to CaW 0.02).

Moreover, Table 2 reveals a significant finding: 9 out of the 13 subjects with CaW consistently exhibit low-velocity regions throughout 100% of the cardiac cycles. In contrast, none of the subjects in the atherosclerosis or normal groups demonstrate stasis throughout all the time frames in the cardiac cycle. This highlights a distinctive pattern unique to individuals with CaW in terms of prolonged low-velocity regions during the cardiac cycle.

## Discussion

Quantifying shear rate, OSI, low velocity regions, and stasis are important in the study of clot formation in patients with CaW. These hemodynamic parameters identify regions of disturbed flow and stagnation downstream from CaW, providing valuable insights into the underlying mechanisms of thrombus formation in vascular disease conditions. Such hemodynamic knowledge is essential for developing interventions and therapies tailored to subjects with CaW. CFD modeling based on patient-specific CTA geometry and PCMR inlet flow conditions was conducted in subjects with CaWs, subjects with mild atherosclerosis with a similar degree of luminal narrowing as CaW subjects, and healthy subjects. The main findings of this study include: (1) subjects with CaW are exposed to larger regions of complex flow represented by low shear rate and high OSI compared to subjects with mild atherosclerosis or normal subjects; (2) CaW patients have larger volumes of low velocity regions; and (3) these low velocity regions represent regions where flow stasis occurs for an extended period of times during the cardiac cycle and particles are not washed out during a single cardiac cycle. *In brief, CaW patients have larger regions of hemodynamics parameters associated with clot formation compared to subjects with atherosclerotic lesions or healthy subjects.*

The results of this study are consistent with what has been seen previously. However, in the current study, we *quantitatively* evaluated hemodynamic parameters associated with clot formation, including OSI, which identifies areas of endothelial disfunction, and low shear/stasis, which is required for clot formation. Blood clot formation can occur through various mechanisms, resulting in distinct compositions of clots depending on their location in the vascular system. Venous clots or 'red clots' form due to prolonged platelet and clotting factor interaction with endothelial cells at low shear rates, leading to coagulation cascade activation in stagnant regions^18^. Thrombi obtained after mechanical thrombectomy associated with CaWs exhibit a high proportion of red blood cells, *i.e.,* ‘red clot’, suggesting formation in low shear rate regions despite limitations in thrombi analysis^19,20,50^. Arterial clots related to other etiologies are associated with high shear rates and platelet aggregation, termed 'white clots,' and are linked to atherosclerosis^33^. High shear rate in subjects with atherosclerosis was observed in this study and it has been well studied in the literature to the high prevalence of white arterial clots^33,51^. The impact of extremely low shear rate of 10s^-1^ on thrombin and fibrin, which are proteins related to blood clot formation has been investigated by Neeves et al.^45^. The study was conducted in membrane-microfluidic channel and it showed that at low shear rate of 10 s^−1^, thrombin penetrated across the entire channel and the fibrin deposited on the membrane took the form of a dense mat of mature fibers^45^. Additionally, another study showed that the a wall shear rate of less than 50 s^−1^ in a venous valve model supported fibrin deposition in the absence of blood cells, depending on multiple factors including Reynolds number and the angle of the valve^35^.

Other studies have investigated the effect of CaW geometry on the local hemodynamic parameters^11,14–16,31^. Bae et al*.*, conducted CFD simulations of artificially generated CaW models, and showed low WSS distal to the web and increased turbulence intensity generated by the web shape in high stenosis and small angles between the web shelf and the vessel wall^14^. Although interesting from a hemodynamics standpoint, the geometries used were not patient-specific geometries. Furthermore, the representation of CaW as an orifice in a diaphragms near the vessel wall especially with stenosis rate > 50% makes the pathophysiologic impact of turbulence intensity in subject with CaW is unknown^14^. Compagne et al., conducted a CFD study based on nine CaW patient-specific geometries and linked CaW to an increase in recirculation zone size^15^. They found significantly higher recirculation area and increased OSI in the CaW bulb compared to the contralateral bifurcation^15^. These results align with the findings of our study, as high recirculation regions were observed in the CaW subjects of this study as well. Another study conducted numerical simulations and computed TAWSS, OSI, RRT and endothelial cell activation potential of modified CaW geometries reconstructed from CTA datasets of eight healthy carotid arteries^52^. The study showed similarly to our results that the webs located at the ICA bulb are more likely to result in disturbed blood flow patterns and thrombus aggregation which may increase the risk of thrombus and subsequent ischemic stroke. A recent study showed that the anatomic and angioarchitecture features of CaW measured using CTA analyzed and a principal component analysis can be used to assess stroke risk^53^. We did not investigate the geometry and the stroke risk in the current study due to limited sample size and all the subjects with CaW had a prior TIA or stroke.

Multiple case reports or small case series have shown that CaW have been linked to thrombus formation and ischemic strokes^17,31^. One study obtained and analyzed a thrombus from a 48-year-old patient with CaW and they performed a CFD simulation using their geometry^31^. Their findings indicate that thrombus formation in CaWs is due to blood stagnation and low WSS. Our study shows similar results showing that subjects with CaWs have lower shear rates compared to mild atherosclerosis and normal subjects, as well as larger stagnation regions continuing for extended periods of time during the cardiac cycles.

Previous work investigated hemodynamics parameters in subjects with CaW using DSA and 4D Flow MRI^11,16^. Park et al., investigated up to 47 carotid arteries with CaW evaluated using DSA and computed the area under the time–density curve as flow stasis evaluation^11^. The study found the amount of flow stasis was significantly higher in CaW compared with mild and moderate carotid atherosclerosis^11^. A prior study utilized 4D Flow MRI to scan subjects with CaW and found that lower TAWSS and higher OSI are present in patients with CaW when compared to patients with atherosclerotic or healthy subjects, which may be linked to clot formation distal to the web^16^. Although 4D flow MRI provides patient specific measurements of the flow field, it has limited spatial and temporal resolution compared to CFD, limiting the ability to quantify hemodynamics distal to the web near the vessel wall^22,54–57^.

The study's results indicate that individuals with atherosclerosis exhibit smaller regions characterized by low shear, high OSI, low velocity, and stasis, in comparison to normal subjects. These findings align with previous studies documented in the literature^16,58^. In subjects with mild atherosclerosis, plaque buildup and vessel wall thickening contribute to blood flow restriction and acceleration, resulting in a high shear rate and low OSI in the ICA bulb^6^. This pattern was observed in this study as well. This suggests the likelihood of a distinctly different clot formation mechanism in individuals with atherosclerosis compared to those with CaW. The expansion of the ICA bulb in the normal carotid bifurcation has been linked to complex blood flow, making it one of the regions in the arterial tree prone to atherosclerosis^59^.

While this study presents valuable insights, it is important to acknowledge certain limitations. One such limitation lies in the assumption of a rigid vessel wall, although this is not expected to be a major source of error given that vessel wall motion is expected to be less than a pixel based on the spatial resolution of the CTA scan^60^. Additionally, our investigation did not account for turbulence, justified by the mild stenosis in all cases and a Reynolds number ranging from 100 to 350. Notably, a higher Reynolds number exceeding 3000 or severe stenosis, is typically required to initiate turbulence. It’s important to highlight that the majority of participants in this study are female. Existing studies in the literature have shown a higher prevalence of CaW in female subjects^3,4,61^. Additionally, subjects with atherosclerosis were older than subjects with CaW, which is due to the fact that’s atherosclerosis usually affects older population. To enhance the robustness of our findings, it is imperative to acknowledge the need for a larger sample size. Expanding the study cohort would enable us to draw more accurate and generalizable conclusions regarding the correlation of stroke risk in patients with CaW and investigate any gender differences. This avenue for future research is essential to deepen our understanding and provide more comprehensive insights into the complex interplay of these variables.

## Conclusion

This study utilized CFD based on patient-specific CTA geometries and PCMR-derived input flow values to quantify hemodynamic parameters in patients with CaW compared to mild atherosclerosis and healthy carotid geometries. CaW was characterized by a statistically significant larger area of complex flow represented by low shear rate, high OSI, low velocity, and higher stasis compared to atherosclerotic lesions and healthy carotids. These results identify hemodynamic parameters to investigate in a large clinical study to predict stroke risk in patients with CaW.

### Supplementary Information

Supplementary Tables.

## Data Availability

The datasets generated during and/or analyzed during the current study are available from the corresponding author on reasonable request.
