# Supplementary material for: Subjects with carotid webs demonstrate pro-thrombotic hemodynamics compared to subjects with carotid atherosclerosis
Source: Sci Rep. 2024 May 2;14:10092. doi: 10.1038/s41598-024-60666-7 (PMC11066020; doi:10.1038/s41598-024-60666-7)
Supplement: Supplementary file 1 — Supplementary Tables. [file 41598_2024_60666_MOESM1_ESM.docx]

# Supplemental Material:

Supplementary Table S1. Shows the CFD simulation parameters computed for each of the subjects averaged across each subject group. The P-value of each parameter was listed below the measurement in round parentheses:

|  | CaW  (p-value vs. atherosclerosis) | Atherosclerosis  (p-value vs. normal) | Normal  (p-value vs. CaW) |
| --- | --- | --- | --- |
| Total Resistance | 21304.2 ± 4965.0  (.88) | 24145.3 ± 8964.5  (.61) | 19943.6 ± 4323.0  (.66) |
| Total Compliance | 0.02 ± 0.01  (.50) | 0.02 ± 0.006  (.67) | 0.03 ± 0.01  (.27) |
| Mean inlet flow | 6.0 ± 1.3  (.84) | 5.6 ± 1.3  (.32) | 6.6 ± 1.4  (.35) |
| Maximum inlet flow | 11.0 ± 2.6  (.77) | 11.8 ± 2.7  (.75) | 12.1 ± 2.6  (.52) |
| Minimum inlet flow | 3.5 ± 0.9  (.11) | 2.5 ± 0.9  (.05) | 4.0 ± 1.2  (.48) |
| delta_T | 0.1 ± 0.05  (.29) | 0.16 ± 0.04  (.23) | 0.1 ± 0.04  (.74) |
| Number of Nodes | 99208.0 ± 30583.1  (.71) | 106615.4 ± 42457.6  (.45) | 123090.5 ± 28246.9  (.22) |
| Number of Elements | 914868.6 ± 1254938.1  (.96) | 607601.4 ± 247866.5  (.46) | 707576.0 ± 168238.9  (.38) |
| Number of Edges | 43531.9 ± 11123.7  (.41) | 48423 ± 15572.8  (.68) | 52072.0 ± 9055.6  (.21) |
| Number of Faces | 29021.3 ± 7415.8  (.41) | 32282 ± 10381.8  (.68) | 34714.7 ± 6037.0  (.21) |
| Time Step Size | 0.002 ± 0.0003  (.68) | 0.002 ± 0.0002  (.68) | 0.002 ± 0.0003  (.95) |
| Simulation Time (hr: min) | 8:32 ± 3:25  (.67) | 9:58 ± 5:57  (.98) | 10:22 ± 4:13  (.71) |

Supplementary Table S2. Shows a complete list of the parameters used to run the CFD simulation in Sim Vascular.

| Parameter | Settings |
| --- | --- |
| Mesh Type | TetGen |
| Boundary Layer | Boundary layers tested 1, 3, 5, and 10.  (Chosen BL = 3.) |
| Grid Independence | Element size tested: 0.04, 0.045, 0.05, 0.06, 0.07. (chosen mesh size 0.05). |
| Cardiac Cycles | Number of cycles Tested: 1-5 cycles.  Number of cycles chosen: 5 cycles. |
| Inlet Boundary Condition | Parabolic velocity profile with flow waveform  MAP as inlet pressure |
| Outlet Boundary Conditions Settings (RCR WindKessel Model) | Capacitance: Stroke Volume/ Pulse Pressure  Resistance: Mean Arterial Pressure/ Mean flow  Murray’s Coefficient: 2.1\|Ratio (Rp: Rd): 1:10 |
| Vessel Wall | Rigid |
| No. of Timesteps | 2400 |
| Time step size | $\frac{No. of cardiac cycles *Cardiac cycle period}{No. of timesteps}$ |
| No. between restarts | 10 |
| Step Construction | 4 |
| Max No. of iterations for NS Solver | 10 |
